# Diagnosis of disorders of consciousness using nonlinear feature derived EEG topographic maps via deep learning

**DOI:** 10.1038/s41598-026-36733-6

**Published:** 2026-02-05

**Authors:** Sheng Qu, Xinchun Wu, Laigang Huang, Yancai Zhou, Qiangsan Sun, Fanshuo Zeng

**Affiliations:** 1 Department of Rehabilitation, The Second Qilu Hospital of Shandong University, No. 247, Beiyuan Avenue, Jinan, 250033 Shandong China; 2 Department of Rehabilitation, Heze Third People’s Hospital, No. 3099, Baji Road, Heze, 274100 Shandong China

**Keywords:** Convolutional neural networks, Disorder of consciousness, Support vector machine, Generalized regression neural networks, Electroencephalography, Nonlinear dynamics analysis, Neurological disorders, Trauma

## Abstract

This study explored the value of nonlinear features extracted from EEG signals to facilitate the assessment of patients with disorders of consciousness (DOC) with limited communication capacity. We utilized a dataset comprising 104 participants, 56 with vegetative state (VS)/unresponsive wakefulness syndrome (UWS) and 48 in a minimally conscious state (MCS). For each participant, we computed channel-wise approximate entropy (ApEn) from EEG time-series data using a sliding window approach under two experimental paradigms: resting state and preferred music stimulation. These nonlinear measures were then spatially interpolated to generate topographical maps. Both resting state and preferred music stimulation data were processed as 1-second epochs using identical convolutional neural networks (CNN) architectures. The classification performance and validity of the CNN were compared against support vector machine (SVM) and generalized regression neural network (GRNN) models. ApEn in the resting state and under stimulation with preferred music correlated with the Coma Recovery Scale-Revised scores in patients with DOC, showing varied regional responses. Notably, the CNNs resulted in a positive diagnostic performance with an accuracy of 90.00% and an AUC of 0.902. The CNN was better than the SVM and GRNN in differentiating between the VS/UWS and MCS states. This study offers a convenient and accurate method for detecting awareness in patients with VS/UWS and MCS using ApEn features in the resting state and under preferred music stimulation using deep learning.

## Introduction

Patients with brain injury may have varying degrees of disorders of consciousness (DOC), even after their lives have been saved^1^. Depending on the degree of preserved consciousness, the main categories include vegetative state (VS)/unresponsive wakefulness syndrome (UWS), in which the eyes open spontaneously without any conscious behavior, and minimal conscious state (MCS), which is characterized by unstable yet reproducible signs of awareness^2^.

The frequency of misdiagnosis of unawareness is as high as 40% when diagnosed solely by clinical consensus without corroboration using behavioral scales^3^. The most sensitive scale used to distinguish between MCS and VS/UWS is the Revised Coma Recovery Scale (CRS-R), which allows longitudinal monitoring of behavioral reactivity in patients with DOC. Error rates can be attenuated by repeated assessment^4^. However, patient factors may mask the true state of consciousness, including cognitive (e.g., aphasia, apraxia) or sensory impairments (e.g., blindness, deafness), minor or easily exhausted motor activity, and pain. In addition, the reliability of clinical assessments is reduced due to the fluctuating response of patients with DOC to instructions or external stimuli^5^. In all these cases, the absence of observed purposeful behaviors at the bedside cannot definitively prove the absence of consciousness. How to accurately estimate the state of consciousness in patients with DOC to help guide optimal healthcare choices and achieve desired patient outcomes remains uncertain.

Neuroimaging is increasingly recognized as helpful in behavioral diagnoses. Notably, the amplitude of the electroencephalography (EEG) signal exhibits marked random fluctuations over time and is non-stationary and nonlinear^6^. In many cases, EEG signals exhibit intermittent repetitions of transient activity rather than sustained oscillations at specific frequencies^7,8^. Entropy-based EEG analysis has received attention in characterizing brain dynamics and has been widely used to assess the “complexity” of EEG in DOC patients^9,10^. We selected approximate entropy (ApEn) as our core metric because it: (1) reliably quantifies signal complexity from brief, noisy recordings^11^, (2) detects functional isolation through decreasing values^12^, and (3) offers clinical practicality with minimal data requirements (100–500 points)^13^. *The ApEn*,* calculated using a sliding-window approach*,* precisely captures local dynamics and has established validity in consciousness monitoring*^7,10,12,14,15^.

Resting-state EEG, which does not require a sophisticated setup or active participation of the subject, has been increasingly used to diagnose DOC^16,17^. However, every state of consciousness is associated with emotions^18^. Auditory stimuli prompt brain activity and form a reliable observational network that helps differentiate between MCS and VS/UWS^19^. The auditory system is less susceptible to damage compared with other parts of the brain after DOC, and is susceptible to fluctuations in states of consciousness^19,20^.

Machine learning techniques may improve the diagnosis of DOC, especially in non-specialist clinics. Deep learning (DL) approaches, specially building convolutional neural networks (CNN), have drawn increasing attention for classification and regression to improve estimation accuracy and robustness. CNN models can directly utilize EEG images as input^21^. CNN-based learning automatically extracts features that alleviate the reliance of traditional approaches on laborious feature engineering, requiring less expertise in domain knowledge and maintaining more information for accurate inference^22^. Specifically, CNNs using time-frequency transforms of EEG data have been used for brain-computer interfaces^23^, detection of focal epileptiform discharges, prediction of outcomes in patients with acute brainstem infarction^24^, and prediction of recovery from coma after cardiac arrest^25^. Nonetheless, the efficacy of CNN models in distinguishing patients with VS/UWS and MCS remains unclear.

This study presents a novel integration of ApEn-derived nonlinear features from both resting-state and auditory stimulation EEG signals within a CNN framework, offering an enhanced approach for DOC diagnosis. It had two main aims. First, we aimed to develop a CNN tool that utilizes ApEn features in a resting state and under auditory stimulation to differentiate between patients with MCS and UWS. Second, to benchmark CNN’s performance against traditional ML methods, we constructed other baseline models using ML algorithms, including a linear support vector machine (SVM) and a generalized regression neural network (GRNN).

## Materials and methods

### Patients

This cross-sectional study recruited 104 participants with DOC who were admitted to the Rehabilitation Department of the Second Qilu Hospital of Shandong university between 17 October 2023 and 10 July 2024. The following inclusion criteria were used: (1) diagnosis of VS/UWS or MCS based on the CRS-R scores; (2) age > 18 years. The following exclusion criteria were used: (1) patients with pre-existing known hearing loss; (2) Unstable state of consciousness characterized by signs of deterioration over the week; (3) diagnosed with locked-in syndrome; (4) electromyography (EMG) artifacts due to severe spasticity; (5) a record of skull fracture; (6) patients with schizophrenia/schizoaffective disorder; (7) the use of sedatives or muscle relaxants within a day before data collection. The Human Subject Ethics Committee of the Second Hospital of Shandong University approved the study protocol. The approval and registration codes are KYLL-2023-414 and ChiCTR2300079310, respectively. Written informed consent was signed by the participant’s family member or legal guardian. The research procedures followed the principles of the Declaration of Helsinki, and all associated patient data were kept confidential.

### Data acquisition

Demographic and clinical characteristics, including sex, age, and days post-injury, were collected from eligible participants. The diagnostic accuracy of EEG was determined using a CRS-R diagnosis obtained through a repeated standardized clinical assessment as a reference^26^. Trained and experienced rehabilitation physicians performed the CRS-R assessment at least once daily for five days. If ambiguity or disagreement persisted between the examiners, the patient was reassessed until the neuropsychological team reached a consensus.

### Experimental paradigm

Initially, EEG signals were recorded in a quiet state for 5 min. Subsequently, music with ‘mood and arousal function’^27^ and ‘autobiographical priming’^28^ capacity was chosen as auditory stimulation to enhance the responsiveness of patients with DOC to external stimuli. EEG signals were recorded for another 5 min while patients listened to their preferred music, which was obtained through interviews with the patients’ family members. To ensure standardized auditory stimulation while minimizing potential artifacts, all preferred music (upbeat and optimistic to prevent emotional bias) was delivered via wireless Bluetooth earbuds with active noise cancellation at 60–70 dB. This wireless setup eliminated cable-related interference and ensured symmetric binaural stimulation, preventing lateralized artifacts that could confound unilateral ApEn analysis. Additionally, EEG data were processed with a 50 Hz notch filter to further suppress any residual line noise.

### EEG recordings

Procedures were performed in a noise-free ward without additional electronic equipment. Data collection was conducted within controlled time windows (8:00–11:30 AM and 1:30–5:00 PM), and patients exhibiting severe drowsiness were excluded. EEG signals were acquired using a wireless 16-channel ZN16E system (Chengdu, China) configured with 19 scalp electrodes positioned according to the 10–20 system (FP1, FP2, F3, F4, C3, C4, P3, P4, O1, O2, F7, F8, T3, T4, T5, T6, FPZ, A1, A2). The configuration employed: (1) bilateral earlobe references (A1-A2), (2) FPZ as ground electrode, and (3) the remaining 16 sites as active recording channels. The signals were digitized at a sampling rate of 500 Hz and a bandwidth of 0.3–100 Hz.

Each participant was assessed in the supine position; a standardized arousal promotion regimen (i.e., deep pressure stimuli from the facial muscle tissue to the toes) was implemented to keep the participant in the wake cycle. *During the EEG recording*,* patients with DOC were asked to relax*,* be quiet*,* wake up*,* and close their eyes.* The entire process was performed with the patients’ eyes closed to maximize EEG data collection while minimizing ocular artifacts.

A rehabilitation physician monitored participants and EEG traces in real-time, identifying potential drowsiness through clinical recognition of prolonged θ bursts (> 3 s duration) and spindle-like waveforms based on visual pattern analysis. The participants were awakened when behavioral and EEG signs of drowsiness appeared. Artifact-free epoch selection was performed offline by an experienced physician through visual inspection of the recordings. Our artifact exclusion protocol consisted of three key steps: (1) Pre-screening exclusion of patients with muscle hypertonia; (2) Intra-recording ocular artifact suppression using light-dampening eye coverings; (3) The physician excluded EEG signals mixed with visible EMG or ocular artefacts, and a stable EEG epoch was recorded (i.e., the noisy portion at the beginning of the recording was discarded). Data were processed using MATLAB software.

ApEn is susceptible to high-frequency components of the EEG signals, and the entire montage is affected by interfering EMG (50–150 Hz). Data with a significant increase in nonlinear metrics throughout the montage were excluded. Finally, each patient with DOC was selected for *60 non-overlapping segments* in the resting state and under auditory stimulation to extract the ApEn topography and values under each electrode for further analysis. Two EEG signal segments (resting state, preferred music), each capturing approximately 32,768 consecutive data points (65.536 s), were selected for further analysis. *For analysis*,* a notch filter (50 Hz) was applied to remove electrical noise*,* a low-pass filter (70 Hz) to reduce myoelectric interference*,* and a high-pass filter (0.3 Hz) to attenuate artifacts.*

### EEG signal extraction

As the EEG time domain is a non-stationary state, effectively representing the irregularity of EEG time series using conventional feature extraction is difficult. Therefore, nonlinear dynamics were used to analyze EEG complexity characteristics.

ApEn^29^ has a robust anti-interference capacity and high stability; minor anomalies do not affect the overall calculation result. With sufficient data, it can be used for both random and deterministic signals and has good generality. As large amounts of EEG data contain both random and deterministic signals, ApEn is suitable for EEG signal extraction. *When applied to EEG signals*,* ApEn quantifies the complexity of neural activity*,* which is a marker of the functional state of the cerebral cortex*^30^. It quantifies the predictability of the subsequent amplitude values of a data sequence based on the knowledge of previous amplitude values. For completely regular data sequences, the knowledge of previous values predicts subsequent values, and the ApEn value is zero. For irregular sequences, the prediction of subsequent values worsens; the approximation increases even if the previous values are known. The chosen parameters included the length of the elapsed time (N), predicted subsequent value (m), and ApEn filtering level (r). To improve the accuracy of the analysis, N was fixed at 4,096. The ApEn was calculated as follows:

1 $$ApEn\,\left( {m,r,L} \right)=\,\frac{1}{{L - m}}\sum \begin{gathered} L - m \hfill \\ i=1 \hfill \\ \end{gathered} \log C_{i}^{{m+1}}\,\left( r \right) - \frac{1}{{L - m+1}}\sum \begin{gathered} L - m+1 \hfill \\ i=1 \hfill \\ \end{gathered} \log C_{i}^{m}\,\left( r \right)$$

In this study, *m* was set to 2, and *r* = 20%standard deviation (SD) of the original time series X_N_. The sliding window length was determined using the sampling length (2s) and was used to perform the ApEn calculations.

ApEn calibrates the degree to which a series of interrelationships quantify a continuum from completely ordered (zero) to completely random (infinite); the larger its value, the more complex or irregular the data. Thus, increasing irregularity (i.e., increasing ApEn) increases nonlinear cellular dynamics or interactions in the cortical network^31^.

### Classifier

#### Two-Way CNN

A CNN typically comprises several layers, including input, convolutional, activation function, pooling, fully connected, and output layers^25,32^. A four-layer convolutional network was used for feature extraction, with each layer comprising 3 × 3 convolutions, batch normalization (to facilitate effective convergence), and a rectified linear unit activation function. The down-sampling process involved 2 × 2 max pooling with a stride of 2. The fully connected layer receives the features extracted from the convolutional network and outputs the final prediction results. The model was trained on a single NVIDIA GeForce RTX A6000 GPU with 48 GB of memory using a batch size of one and training for 500 epochs. An RMSProp optimizer was employed with a learning rate of 0.01. The training parameters, including alpha = 0.99, weight decay = 0, momentum = 0, and epsilon = 1 × 10 − 8, were set to default values established in the literature^33^. *From each patient’s recordings*,* three EEG images were generated for each of the two conditions (resting state and preferred music)*, with 20 EEG topographies on each image, i.e., each patient ended up with 6 EEG images (120 topographies) that were incorporated into the CNN model. The patient’s EEG images, originally at a 512 × 512 RGB resolution, were cropped to 256 × 256 pixels to address memory consumption concerns. Below is the detailed layer-wise structure in Table 1:

**Table 1:** Layer-wise architecture of the Two-Way CNN.

Layer	Operation	Kernel/Stride	Output Shape	Trainable Parameters
Input	-	-	256 × 256 × 3	0
Conv1 + BN + ReLU	3 × 3 convolution	3 × 3 / 1	256 × 256 × 32	896
MaxPool1	2 × 2 max pooling	2 × 2 / 2	128 × 128 × 32	0
Conv2 + BN + ReLU	3 × 3 convolution	3 × 3 / 1	128 × 128 × 64	18,496
MaxPool2	2 × 2 max pooling	2 × 2 / 2	64 × 64 × 64	0
Conv3 + BN + ReLU	3 × 3 convolution	3 × 3 / 1	64 × 64 × 128	73,856
MaxPool3	2 × 2 max pooling	2 × 2 / 2	32 × 32 × 128	0
Conv4 + BN + ReLU	3 × 3 convolution	3 × 3 / 1	32 × 32 × 256	295,168
MaxPool4	2 × 2 max pooling	2 × 2 / 2	16 × 16 × 256	0
Flatten	-	-	65,536	0
Dense	Fully connected	-	1	65,537

**Abbreviations**: Total Parameters: 453,95, BN: Batch Normalization, ReLU: Rectified Linear Unit.

#### GRNN

Specht described generalized regression neural networks (GRNN) as probabilistic neural networks^34^. Similarly, in multilayer error backpropagation neural networks, GRNNs can approximate any functional relationship between the inputs and outputs under appropriate conditions. Specifically, GRNNs can be used as classifiers to categorize test samples into two or more classes. This algorithm is not prone to local minima, requires fewer tuning parameters for optimization, and can be used to analyze large and unstable datasets^35^. The GRNN-specific algorithms used here were based on our previous research^36^. The GRNN was built using an in-house software program in MATLAB (version R2021a, MathWorks, Natick, MA, USA).

#### SVM

A support vector machine (SVM) is a supervised learning algorithm used for binary and multiple classification problems. It classifies items by finding an optimal hyperplane in the feature space with maximum intervals. This study used the SVM toolbox function fitcsvm provided in MATLAB. The features and labels of the input function samples were used for model training. The input sample dimensions were 76 × 15, where 76 represents the number of samples and 15 the number of features.

#### Model performance

*The classification performance was evaluated using the area under the receiver operating characteristic curve (ROC-AUC). An AUC value > 0.60 was considered to indicate enhanced classification ability.* Delong’s test was used to assess the statistical significance of the differences between the ROC curves of the three models. Based on the predicted results and the actual labels of the samples, they can be categorized into four types: true positive (TP), false positive (FP), true negative (TN), and false negative (FN). The confusion matrix for binary classification was then obtained, as shown in Table 1. Sensitivity, specificity, and accuracy can be derived from the confusion matrix. The corresponding formulae are as follows:

2 $$Accuracy=\frac{{TP+TN}}{{TP+TN+FP+FN}}$$

3 $$Sensitivity=\frac{{TP}}{{TP+FN}}$$

4 $$Specificity=\frac{{TN}}{{TN+FP}}$$

5 $$\Pr ecison=\frac{{TP}}{{TP+FP}}$$

6 $$F1=\frac{{2*TP}}{{2TP+FP+FN}}$$

### Statistical analyses

Data analyses were performed using IBM SPSS for Windows (version 26.0; IBM Corp., Armonk, NY). The Shapiro–Wilk test was used to assess the distribution normality for age, days post-injury, and total CRS-R score. Summary statistics for normally distributed data are presented as mean and standard deviation (SD), whereas summary statistics for non-normal data are presented as interquartile range (IQR). The independent samples *t*-test and Mann–Whitney *U* test were used to compare normal and non-normal data. Categorical variables are presented as percentages. Correlation analysis of the mean ApEn on the 16 electrodes with the total CRS-R score was performed using Spearman’s correlation analysis, with *r* indicating the strength of the correlation. The preferred music-induced ApEn values for patients with VS/UWS and MCS were compared using a one-way ANOVA with Bonferroni correction. The significant variables from the univariate analysis were incorporated into the SVM and GRNN classifiers. Figures were generated using GraphPad Prism version 6.01 (San Diego, CA, USA). Statistical significance was set at *P* < 0.05.

## Results

### Baseline patient characteristics

**Fig. 1:**
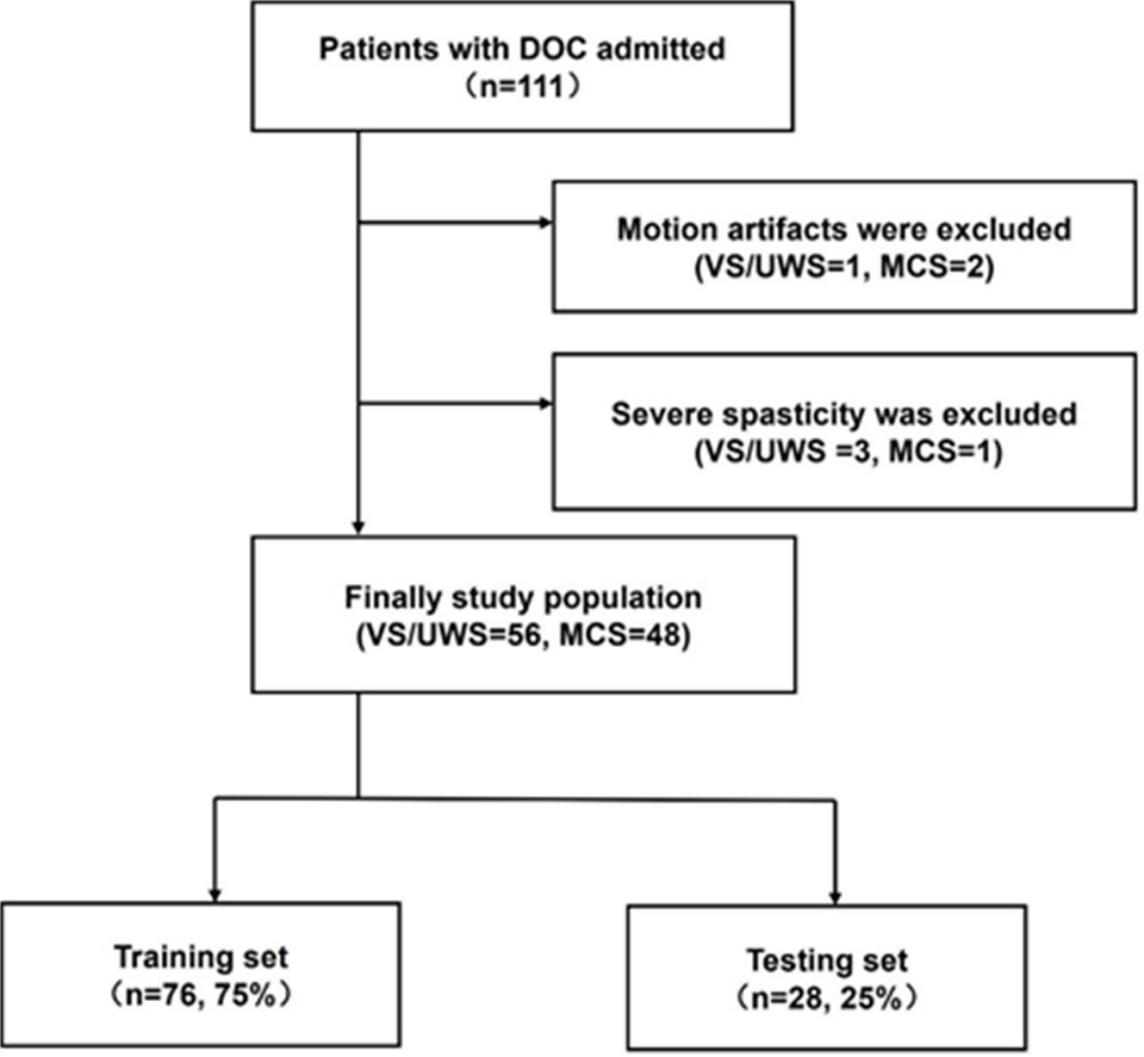
Patient selection process. ApEn: approximate entropy, CNN: convolutional neural network, EEG: Electroencephalogram, MCS: minimally conscious state, VS/UWS: vegetable state/unresponsive wakefulness syndrome.

**Fig. 2:**
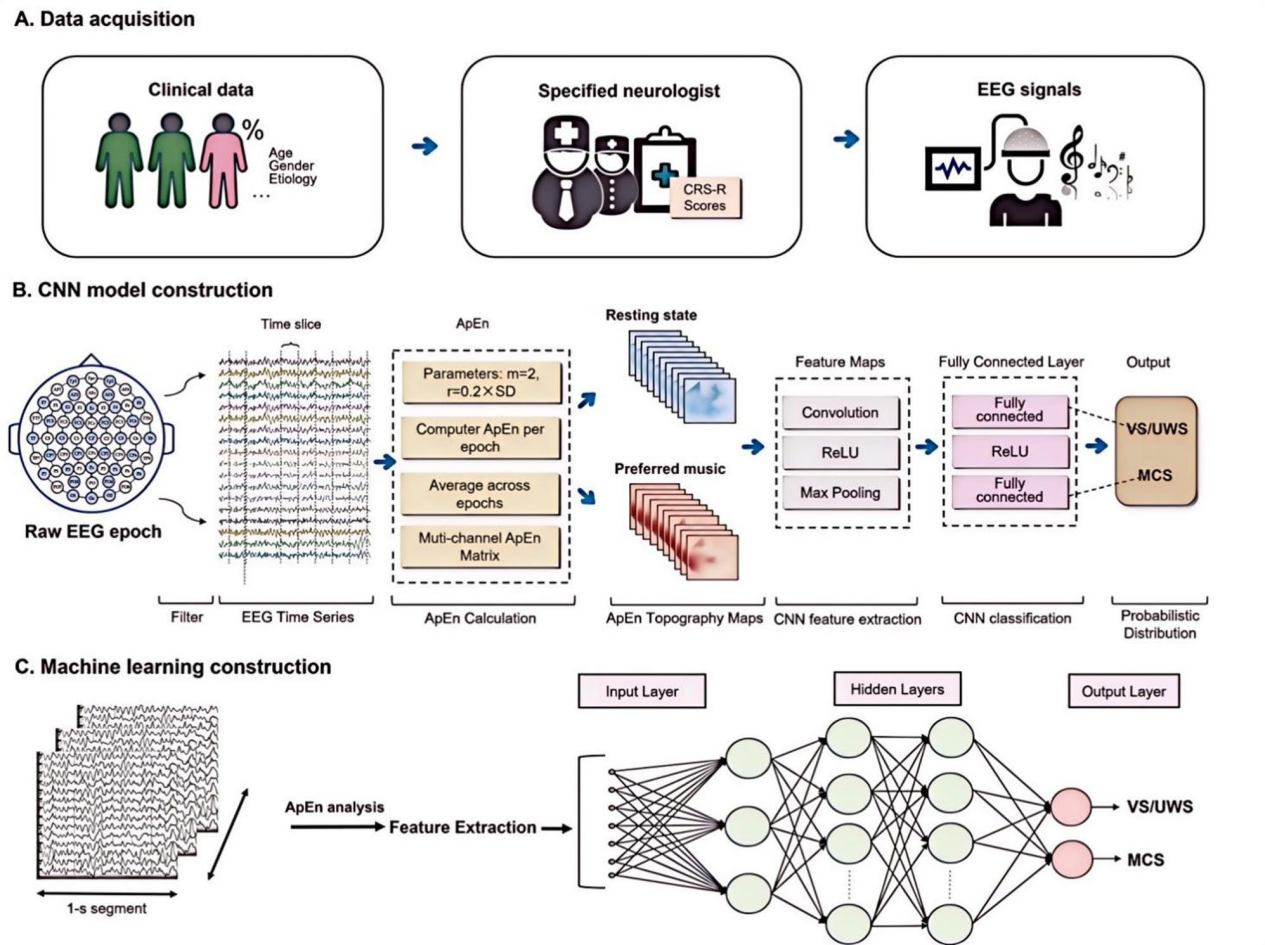
*Schematic diagram of the computational framework*. (**A**) Participant enrolment, clinical evaluation, and scanning procedures. (**B**) Construction CNNs. (**C)** Construction of traditional machine learning frameworks, i.e., SVM and GRNN. ApEn: approximate entropy, CNN: convolutional neural network, EEG: Electroencephalogram, MCS: minimally conscious state, VS/UWS: vegetable state/unresponsive wakefulness syndrome.

The patient selection process is illustrated in Fig. 1. A total of 111 patients with DOC were initially screened; of these, 3 were excluded because of motion artefacts and 4 due to severe spasticity. Ultimately, 104 patients were included, of which 76 served as the training set; 28 served as the test set, and were used to evaluate the performance of the classifier model. The demographic information of the training and test sets is presented in Table 2. In the training set, patients in VS/UWS and MCS were 69.23% (*n* = 27) and 67.57% (*n* = 25) male, respectively, and had a median age of 60 and 69 years, duration post-injury of 76 and 75 days, CRS-R total scores of 4 and 9, and CRS auditory scores of 1 and 2. However, in the training set, significant differences existed between the VS/UWS and MCS groups in terms of injury location, with bilateral/diffuse damage in patients with MCS (*P* < 0.05). Overview of the diagnosis of the framework in Fig. 2.

**Table 2:** Distributions of characteristics at baselines between the training and test sets.

	Training set (*n* = 76)		Test set (*n* = 28)	
	VS/UWS (*n* = 39)	MCS (*n* = 37)	VS/UWS (*n* = 17)	MCS (*n* = 11)
Age, days (IQR)	60.00 (47.00, 72.00)	69.00 (51.50, 76.50)	57.24 ± 14.84	58.09 ± 17.57
Days post-injury, days (IQR)	76.00 (48.00, 154.00)	75.00 (46.50, 190.00)	118.00 (30.50,282.50)	149.00 (74.00, 359.00)
Male, *n* (%)	27 (69.23%)	25 (67.57%)	11 (64.71%)	9 (81.81%)
Etiology, *n* (%)				
Trauma	7	8	1	4
Stroke	30	27	13	6
Anoxia	2	1	3	1
	0	1		
Injury location, *n* (%)				
Unilateral	**10** ^a^	19	9	6
Bilateral/diffuse	29	18	7	5
CRS-R auditory scores, median (IQR)	1 (1, 2)	2 (2, 3)	1 (1, 1)	3 (2, 3)
CRS-R total scores, median (IQR)	**4 (2**,** 5)** ^**b**^	9 (8,10)	**5 (2**,** 6)** ^**b**^	8 (6,10)

**Abbreviations**: IQR: interquartile range, CRS-R: Revised Coma Recovery Scale. ^a^Chi-square test found a significant difference. ^b^One-way ANOVA followed by Mann–Whitney U.

### Association of EEG features with prognosis and clinical behavior

**Fig. 3:**
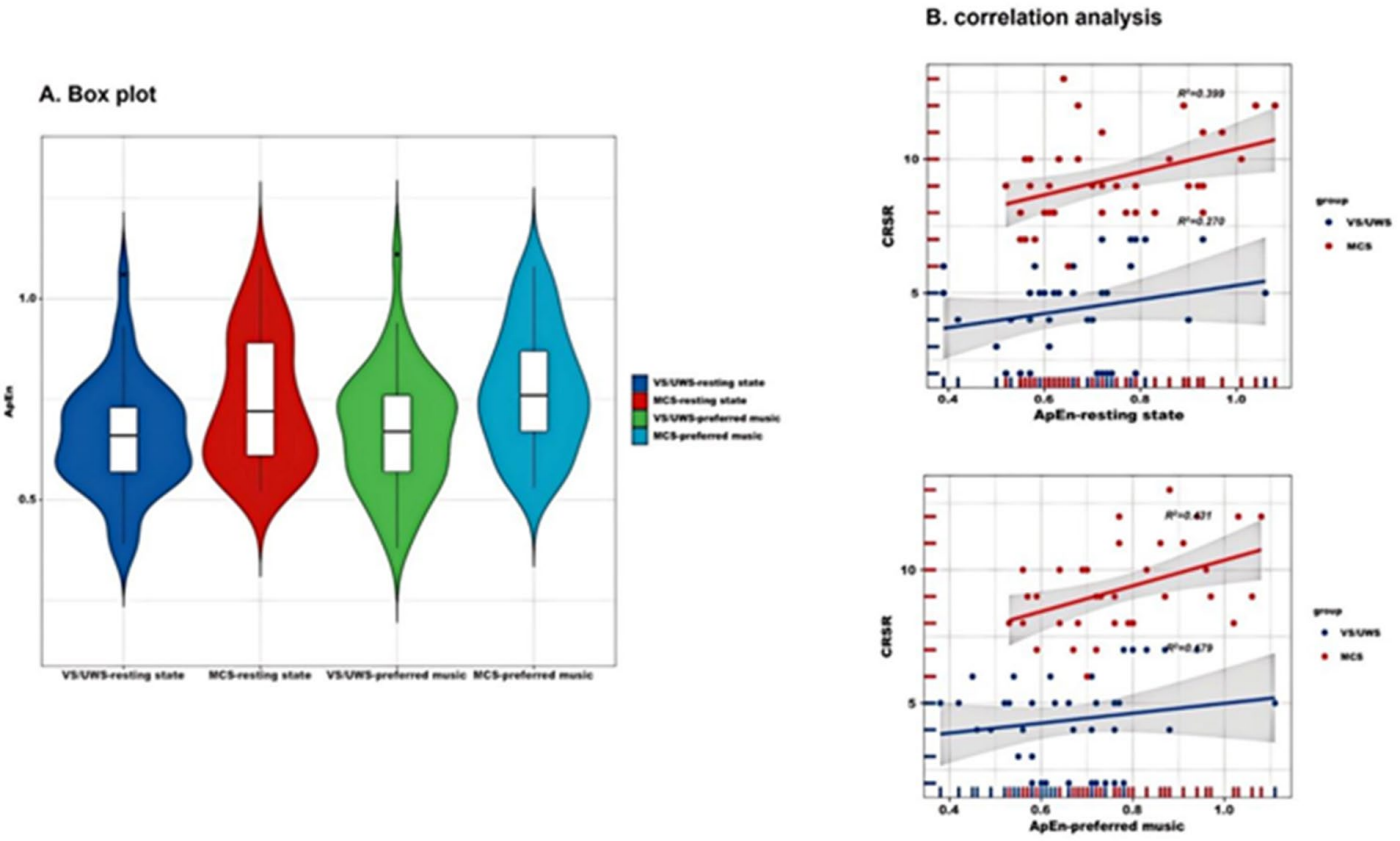
Violin plot and correction analysis. (**A**) Distribution of ApEn in VS/UWS and MCS patients in the resting state and under stimulation with preferred music. (**B**) Correlation between CRS-R total score and ApEn means in VS/UWS and MCS patients. ApEn: approximate entropy, CNN: convolutional neural network, EEG: Electroencephalogram, MCS: minimally conscious state, VS/UWS: vegetable state/unresponsive wakefulness syndrome.

Violin plots of the distribution of ApEn values in patients with DOC in the resting state and in the preferred music are shown in Fig. 3A. We performed correlation analyses to identify the relationship between clinical variables and ApEn values in patients with DOC. The results of the correlation analysis are shown in Fig. 3B. The mean value of ApEn on the electrodes was not significantly correlated with the total CRS-R scale score in patients with VS/UWS, either in a quiet state or with their preferred music (*P* > 0.05). Moreover, the mean ApEn values of patients with MCS in preferred music were positively correlated with the total CRS-R score, with a correlation coefficient of 0.431 (*P* = 0.008). The mean value under the ApEn electrode was positively correlated with the total CRS-R score in MCS patients in the resting state (*R* = 0.399, *P* = 0.014).

### Machine learning variable screening

We investigated the association between ApEn values and the state of consciousness (VS/UWS and MCS) in both paradigms (Table 3). In the resting state, patients with MCS had significantly higher ApEn values at FP1, F3, C3, P3, F7, T3, and T5 than did VS/UWS patients (*P* < 0.05, one-way ANOVA). With preferred musical stimulation, the ApEn values in FP1, F3, C3, P3, O1, F7, T3, and T5 were significantly higher in patients with MCS than in patients with VS/UWS (*P* < 0.05, one-way ANOVA). Thus, 15 variables were included in the SVM and GRNN models.

**Table 3:** Mean ApEn values to each condition and group by individual electrodes.

Montage	Resting-state				Preferred music			
	VS/UWS	MCS	F	*P*-value	VS/UWS	MCS	F	*P*-value
**FP1**	0.60 ± 0.14	0.72 ± 0.18	9.96	**0.002**	0.59 ± 0.17	0.77 ± 0.18	18.92	**<0.001**
**FP2**	0.61 ± 0.17	0.65 ± 0.17	0.96	0.332	0.64 ± 0.18	0.66 ± 0.18	0.43	0.516
**F3**	0.68 ± 0.19	0.78 ± 0.24	5.08	**0.027**	0.70 ± 0.22	0.82 ± 0.24	5.89	**0.018**
**F4**	0.66 ± 0.15	0.74 ± 0.21	3.50	0.066	0.68 ± 0.18	0.75 ± 0.18	3.01	0.087
**C3**	0.62 ± 0.18	0.77 ± 0.24	10.22	**0.002**	0.62 ± 0.17	0.81 ± 0.24	15.93	**<0.001**
**C4**	0.68 ± 0.17	0.75 ± 0.20	2.33	0.131	0.70 ± 0.16	0.76 ± 0.18	2.67	0.107
**P3**	0.67 ± 0.19	0.79 ± 0.19	7.62	**0.007**	0.68 ± 0.17	0.82 ± 0.18	11.96	**0.001**
**P4**	0.64 ± 0.19	0.71 ± 0.22	2.35	0.129	0.66 ± 0.21	0.73 ± 0.22	2.36	0.129
**O1**	0.67 ± 0.18	0.74 ± 0.19	2.93	0.091	0.67 ± 0.18	0.78 ± 0.20	6.13	**0.016**
**O2**	0.67 ± 0.18	0.75 ± 0.20	0.07	0.066	0.70 ± 0.19	0.78 ± 0.19	2.89	0.093
**F7**	0.62 ± 0.21	0.72 ± 0.21	4.36	**0.040**	0.63 ± 0.22	0.77 ± 0.22	7.27	**0.009**
**F8**	0.70 ± 0.19	0.75 ± 0.19	1.64	0.205	0.71 ± 0.19	0.78 ± 0.19	2.62	0.110
**T3**	0.66 ± 0.20	0.76 ± 0.20	4.41	**0.039**	0.66 ± 0.20	0.79 ± 0.18	8.44	**0.005**
**T4**	0.70 ± 0.18	0.73 ± 0.23	0.58	0.450	0.72 ± 0.19	0.78 ± 0.22	1.94	0.168
**T5**	0.64 ± 0.19	0.75 ± 0.20	5.90	**0.018**	0.66 ± 0.20	0.78 ± 0.19	7.87	**0.006**
**T6**	0.71 ± 0.19	0.74 ± 0.22	0.25	0.617	0.73 ± 0.19	0.78 ± 0.16	1.78	0.187

**Abbreviations**: ApEn: approximate entropy, CNN: convolutional neural network, MCS: minimally conscious state, VS/UWS: vegetable state/unresponsive wakefulness syndrome, SVM: support vector machine, GRNN: generalized regression neural network.

### Model performance in the validation set

**Fig. 4:**
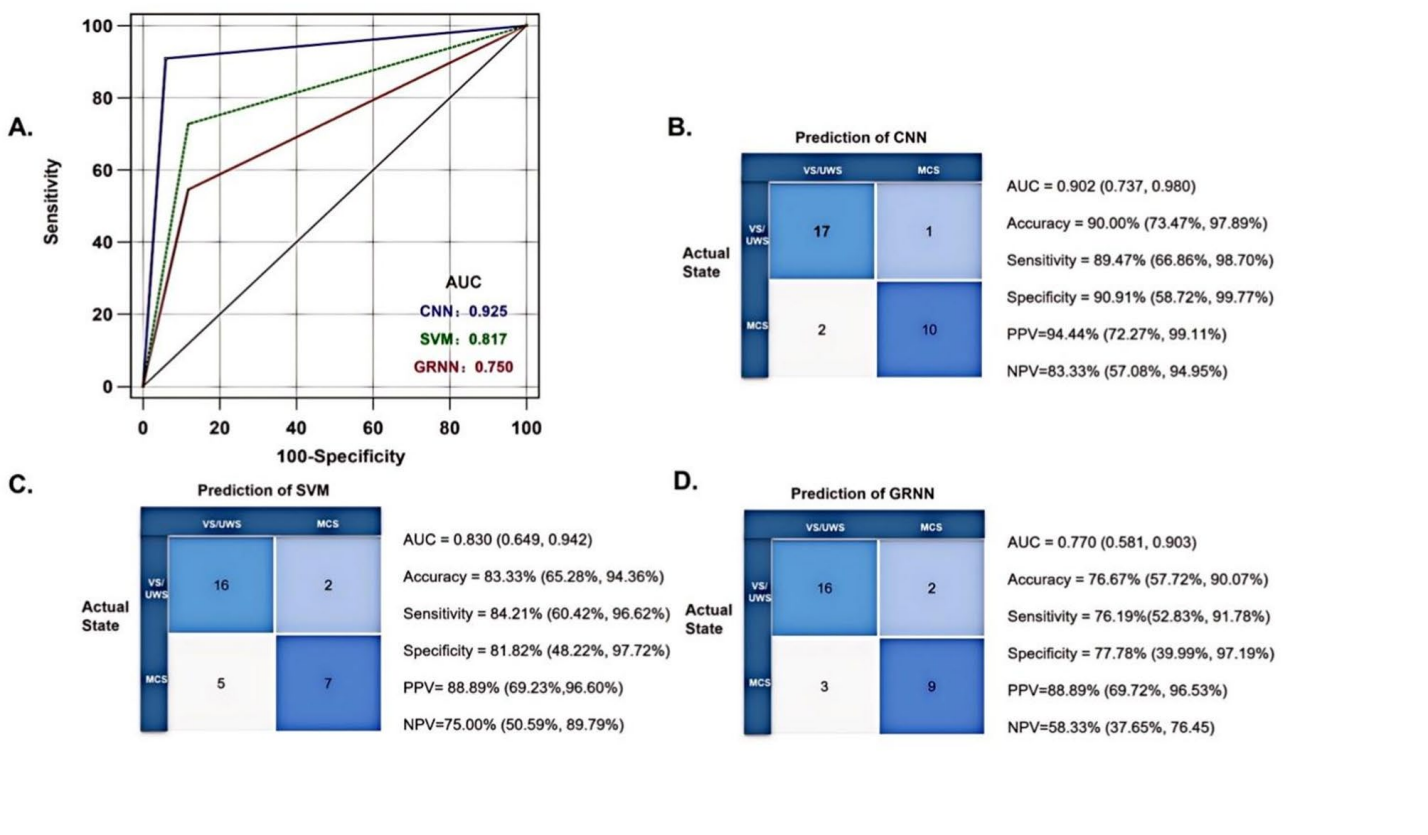
Confusion matrices and ROC curves. ApEn: approximate entropy, CNN: convolutional neural network, EEG: Electroencephalogram, MCS: minimally conscious state, VS/UWS: vegetable state/unresponsive wakefulness syndrome.

The comparative performance of all classifiers in the validation set is summarized in Table 4; Fig. 4 (showing confusion matrices and ROC curves). The CNN achieved numerically higher metrics than SVM, including AUC (0.902 vs. 0.830, *P* > 0.05 by DeLong test). While this difference was not statistically significant, both CNN and SVM significantly outperformed GRNN in AUC (*P* < 0.05 by DeLong test). Complete performance metrics with 95% CIs are tabulated in Table 4.

**Table 4:** Performance comparison of machine learning.

Model	AUC	Accuracy	Sensitivity	Specificity	PPV	NPV
**CNN**	0.902 (0.737, 0.980)	90.00% (73.47%, 97.89%)	89.47% (66.86%, 98.70%)	90.91% (58.72%, 99.77%)	94.44% (72.27%, 99.11%)	83.33% (57.08%, 94.95%)
**SVM**	0.830 (0.649, 0.942)	83.33% (65.28%, 94.36%)	84.21% (60.42%, 96.62%)	81.82% (48.22%, 97.72%)	88.89% (69.23%,96.60%)	75.00% (50.59%, 89.79%)
**GRNN**	0.770 (0.581, 0.903)	76.67% (57.72%, 90.07%)	76.19% (52.83%, 91.78%)	77.78% (39.99%, 97.19%)	88.89% (69.72%, 96.53%)	58.33% (37.65%, 76.45)

**Abbreviations**: AUC: Area under the curve, PPV: Positive Prediction Value, NPV: Negative Prediction Value.

## Discussion

DOCs are often caused by brain lesions in individuals, resulting in similar states of unconsciousness. Given that selecting and managing an appropriate rehabilitation program requires awareness, an objective quantitative classification method for patients with DOC is urgently needed. Our study had several key findings. First, using EEG images from the resting state and preferred music, the CNN classified VS/UWS and MCS with an accuracy of 90.00% and an AUC of 0.902. Second, the CNN outperformed the SVM and GRNN in all evaluation metrics. Third, the ApEn of the resting state and preferred music correlated with behavioral CRS-R scores in patients with DOC, suggesting that the patients’ states of consciousness were highly synchronized with the ApEn.

The availability and robustness of EEG make it a promising tool for bedside diagnostic evaluation of patients with DOC, at least as a first-line/screening diagnostic procedure. Our team has conducted previous research showed a positive correlation between elevated mean ApEn values and CRS-R scores, i.e., ApEn values were higher in the MCS than in the VS. Emotion or familiarity attached to a stimulus reportedly elicits a stronger response than a neutral stimulus^37^; therefore, preferred music is more conductive to elicit the expression of residual cognitive functioning in the diagnosis of DOC. In a prognostic study of DOC patients^38^, the ‘task’ paradigm had lower sensitivity but higher specificity than the ‘resting state’ paradigm. Therefore, combining different EEG paradigms in clinical practice can potentially improve DOC classification accuracy. Emotional salience and autobiographical context may be important for accurately assessing residual cognitive ability^15,28,39^.

Resting-state analyses revealed significantly higher ApEn in left cortical regions among MCS patients versus VS/UWS patients (*p* < 0.05). Notably, these findings align with established neural correlates of consciousness, which demonstrate: (1) strengthened functional connectivity between the posterior cingulate cortex (default mode network) and left anterior insula (salience network); (2) elevated fractional amplitude of low-frequency fluctuations in the left prefrontal executive control network (ECN); and (3) a positive association between left ECN activity and behavioral responsiveness (CRS-R scores: *r* = 0.34, *p* = 0.04)^40^. Furthermore, left occipital glucose metabolism (CMRGlu) was markedly higher in MCS patients (*P* = 0.013), suggesting residual environmental awareness may depend on preserved left occipital function^41^. Importantly, our finding of left-lateralized ApEn patterns, particularly enhanced left-hemispheric signal complexity in MCS versus VS/UWS patients during music stimulation-further implicates left-hemispheric network integrity in minimal consciousness. This observation aligns with existing evidence that low-frequency music selectively activates the left prefrontal cortex and primary somatosensory cortex (S1) in MCS patients^42^. In stark contrast, VS/UWS patients typically demonstrate diffuse structural damage to thalamocortical and ascending reticular activating system (ARAS) pathways, which may account for their impaired network activation patterns and reduced responsiveness to sensory stimulation.

While ML in medicine has shown a remarkable potential to improve the diagnosis and prognosis of various neurological disorders. However, its application in diagnosing DOC remains limited. Notably, DL replaces the traditional complexity of manual feature extraction and avoids the influence of ML on the a priori knowledge of the results, allowing for the retention and processing of complex information and finding more accurate links between inputs and outputs^43^. Supporting this, Aellen et al. ^25^ developed a CNN model in their 2023 *Brain* study that analyzed early EEG patterns to predict 3-month awakening in comatose post-cardiac arrest patients undergoing target temperature management (TTM; 33 °C/36°C), demonstrating robust performance (PPVs: 0.83 − 0.81; AUCs: 0.69–0.70). Similarly, another study reported an AUC of 0.885 using one-dimensional CNNs^44^. Building on these developments, our CNN model attained 90.00% accuracy in ApEn-based EEG classification for DOC, a result comparable to the 87.6% accuracy achieved by Pan et al. ^45^ using self-supervised contrastive domain generalization (SSCDG). Notably, Pan et al.‘s framework enables knowledge transfer from healthy to DOC subjects through self-supervised learning, further advancing the field. Recent progress demonstrates the superiority of multiscale CNNs with few-shot learning (e.g., Cai et al.‘s^46^ MSCNN-FSL), which outperforms conventional single-scale approaches by achieving > 64% accuracy while mitigating overfitting in small datasets. Furthermore, DL applications continue to show advantages over traditional ML, as evidenced by Huan et al.‘s ^47^ DeepDOC (AUC = 0.927, accuracy = 0.861) for rs-fMRI classification. While our study confirms that traditional ML performs adequately in DOC diagnosis, DL algorithms maintain a clear performance advantage.

Notably, our model significantly outperformed the SVM (discrimination score: 0.84) and GRNN baselines (*P* < 0.05), underscoring the potential of simpler architectures when combined with ApEn feature engineering. SVM is a supervised learning algorithm popular in DOC research as a kernel-based classification method with built-in mechanisms for controlling overfitting tendencies. Liang et al.^48^ constructed a multidimensional EEG nonlinear metric model to discriminate between two-dimensional consciousness using a genetic algorithm-based SVM and observed (AUC = 0.923) that it outperformed BP (Backpropagation) and RF (Random Forest) Neural Networks. Another study trained an SVM classifier using auditory-induced absolute power spectral density differences to predict DOC prognosis with an accuracy of 0.727 and an average AUC of 0.877^18^. Diffusion tensor imaging combined with an SVM revealed that thalamic tracks reaching the frontal, parietal, and sensorimotor regions could discriminate VS, MCS^−^, and MCS^+^ across each region, with up to 100% accuracy^49^. Our previous study found that, based on clinical indicators, GRNN performed well in predicting the patient’s prognosis^36^. Therefore, this study attempted to combine a GRNN with extracted EEG data features to diagnose DOC; however, its performance was lower than that of the SVM. Some studies have used GRNN to construct EEG-based classification models, mainly for epilepsy classification^50^ and recognition of control artifact tasks^34^; these perform relatively well. Overall, this study’s SVM and GRNN applications performed less well than the CNN in classifying DOC. We hypothesized that in DOC, the uncertainty of traditional ML feature extraction, owing to unknown pathogenic mechanisms and insufficient a priori knowledge, may help explain the poor results.

This study has some limitations: First, this study only explored the diagnostic performance of ML models in VS/UWS and MCS states with a small sample size. Further studies should focus on more specific subgroups of patients-particularly Minimally Conscious State plus (MCS+), Minimally Conscious State minus (MCS-), and Atresia Syndrome—increase patient sample size, and improve methods for feature selection. Second, although patients are assessed in an ‘awake’ state, some VS/UWS patients are unable to fully monitor the patient’s state of alertness during data acquisition due to the lack of a sleep-wake cycle. Therefore, the possibility that the patient fell asleep during the scanning process cannot be ruled out, which may affect the accuracy of the test results. Third, while our CNN model’s supervised learning framework demonstrates strong classification performance, its generalizability across inter-subject EEG variability may be constrained. Similarly, the current ApEn-based feature extraction, though computationally efficient, could be enhanced to better capture domain-invariant neural patterns. Building on recent advances in few-shot learning, future work will: (1) integrate multiscale convolutional architectures (MSCNN-FSL) to improve cross-subject generalization through hierarchical feature learning, and (2) implement self-supervised contrastive domain generalization (SSCDG) frameworks to enhance robustness against distribution shifts while reducing annotation dependence. Fourth: This preliminary study demonstrates the feasibility of CNN-based analysis with single-metric ApEn EEG topography in a small exploratory cohort, highlighting the potential of nonlinear EEG features for DOC assessment. *Future research should focus on the multimodal integration of complementary nonlinear measures-Lempel-Ziv Complexity*,* Correlation Dimension*,* and Largest Lyapunov Exponent-to develop more comprehensive diagnostic models.* Finally, the CNN’s 3 × 3 convolutional kernels emulate local receptive fields, yet the model’s decisions remain neurophysiologically opaque. While batch normalization improves training, techniques like Grad-CAM are ultimately required to localize clinically meaningful brain regions.

## Conclusion

This study showed strong links between the ApEn characteristics of the EEG response (resting state, preferred music) and consciousness states, reflecting neural synchrony and complexity, which have been previously associated with the presence of consciousness. Given the heterogeneity of DOC pathophysiology, CNNs can retain and process complex information, allowing them to classify VS/UWS and MCS more accurately in an automated and user-independent manner. This result may also explain the superiority of CNNs over traditional ML. Our findings call for a more systematic use of auditory stimuli in clinical routines, combined with state-of-the-art DL algorithms, to assist and diagnose patients with DOC in the grey area of consciousness.

## Data Availability

Data supporting the results of this study are available on request from the corresponding authors. This data will not be made public due to privacy or ethical restrictions.
